# Presence of Borrelia Spirochetes in White Stork (*Ciconia ciconia*), White-Tailed Eagle (*Haliaeetus albicilla*), and Eastern Imperial Eagle (*Aquila heliaca*): Hospitalized in a Wild Bird Hospital and Sanctuary (Hortobágy, Hungary)

**DOI:** 10.3390/ani14243553

**Published:** 2024-12-10

**Authors:** András Pál Bózsik, János Déri, Béla Pál Bózsik, Borisz Egri

**Affiliations:** 1 Department of Animal Science, Albert Kázmér Faculty of Agricultural and Food Sciences, Széchenyi István University, 9200 Mosonmagyaróvár, Hungary; egri.borisz@sze.hu; 2 Bird Hospital Foundation, 4071 Hortobágy, Hungary; 3 Lyme Diagnostics Ltd., 2011 Budakalász, Hungary

**Keywords:** *Borrelia anserina*, *Borrelia burgdorferi*, migratory birds, birds of prey, human pathogenic, vector-borne, reservoir

## Abstract

Wild birds across the globe can carry the causative agent of avian borreliosis, *Borrelia anserina*, and that of human Lyme borreliosis, *Borrelia burgdorferi* sensu lato in the ticks attached to them. Currently, only limited proof exists for the presence of these pathogens in samples taken from living wild birds, carried by the birds as a reservoir, without symptoms. We investigated blood samples of large-bodied wild birds admitted to a bird hospital, where basic clinical symptoms were recorded. Thirty-six blood samples were collected from them, according to the DualDur kit methodology, and investigated using dark-field microscopy to search for spirochetes. Five of the samples were also investigated by immunofluorescence microscopy to identify *Borrelia burgdorferi*. Since the main reason for admittance to the hospital was a physical injury, no significant symptoms were observed regarding a current Borrelia infection. Out of the sixteen birds of prey, eleven (68%) were infected with spirochetes, and fifteen out of twenty storks (75%) were also infected with spirochetes, without major symptoms. All five samples investigated using immunofluorescence were confirmed to contain the human pathogenic *Borrelia burgdorferi* sensu lato. Thus, direct investigations of the blood of wild birds may show the asymptomatic prevalence of Borrelia in the wild bird population.

## Simple Summary

Wild birds fly across countries and continents. They carry pathogens in their blood, and they might also carry infected parasites like ticks or lice. Their flight path spreads diseases over long distances. Some birds may carry pathogens that are not infectious to the birds themselves, only to humans, in which case they are reservoirs for these bacteria. Bacteria are often spread from reservoirs to humans by blood-sucking insects like ticks. These bacteria, especially the spiral-shaped Borrelia spirochetes, are usually not detected via direct blood investigation, like microscopy, because they are low in numbers, so concentration steps are necessary. In this research we investigated bacteria extracted from 36 blood samples collected in a wild bird hospital from wild birds that did not show specific symptoms for a Borrelia infection. Still, at least two thirds of the samples contained Borrelia (e.g., *B. anserina*, *B. burgdorferi* sensu lato). All five samples subjected to the specific investigation confirmed the presence of the human-pathogenic *Borrelia burgdorferi*, which proves that not only are ticks carried by wild birds but the blood of birds can also potentially transmit human diseases.

## 1. Introduction

The blood of birds may contain various disease agents: viruses, bacteria, rickettsiae, protozoa, microfilariae, and fungi. Free-living birds constitute potential for intra-species and inter-species transmission of bacterial infections while being affected by the diseases, or they can be reservoirs or sentinels and even potentially spread antibiotic-resistant pathogens. Some infections are spread in the intestines, e.g., *Salmonella*, *Campylobacter*, *Yersinia enterocolitica*, *Clostridium*, *Escherichia coli*, *Vibrio*, and *Listeria*. Some bacteria constitute the normal or unhealthy flora of the skin or mucous membrane of wild birds, e.g., *Staphylococcus aureus* and *Yersinia pseudotuberculosis* [1]. Out of all these bacteria, some can even be detected in the blood and may cause septicemia, e.g., *S. aureus*, *L. monocytogenes*, and *Y. pseudotuberculosis*. However, true blood-borne pathogens are rarely described in wild birds.

Among blood-borne pathogens, one of the rarely studied areas is the presence of spirochetes, especially the Borrelia species in wild birds, and these birds, besides carrying infected ticks, may contribute to the spreading of diseases across continents within their bodies.

The specialized pathogen of birds, *Borrelia anserina*, compared to other Borrelia species, progresses faster and causes a more severe disease in the host, with surviving body temperatures exceeding 42 °C. An important fact from a biodiversity and economic point of view is that it leads to the death of the fowl, in up to 20% of cases, whereas the surviving individuals acquire effective immunity [2]. Other Borrelia species do not necessarily cause immunity that completely prevents reinfection or reactivation [3,4]

Avian borreliosis, caused by *Borrelia anserina* is an acute septicemic disease in a wide range of avian species. In many countries worldwide, this infection used to cause severe disease resulting in the loss of a large proportion of poultry at the industrial sites [2], mainly those with poor sanitation and grown in free-range conditions. The disease has a very low prevalence in large intensively managed flocks of domestic birds due to the good management of its vector, *Argas* spp. (soft) ticks, and it is now considered confined to small flocks kept for family consumption [5]. Nevertheless, there is still evidence of the worldwide presence of *B. anserina*. Thus, wild birds as a reservoir of *B. anserina* still need to be studied more in-depth, including the genetic sequencing of its various substrains. For example, new species of recurrent fever type borreliae, the same family of Borreliae that *B. anserina* belongs to, were found in ticks feeding on various avian hosts in the US, in which the genome sets of borreliae were 85–96% identical to known *B anserina* species in the gene bank [6]. *Borrelia anserina* is transmitted by species of ticks with a worldwide presence; thus, a recent paper from 2022 concludes that the real geographical distribution is vastly underestimated if we only consider the few pieces of experimental evidence currently available [7]. Avian spirochetosis is concurrent with the presence of *Argas* spp. ticks, which serve as both a reservoir and the primary vector. These ticks even feed on mammals, such as bats, while carrying the *B. anserina* spirochetes [8]. Proven infections with *B. anserina* also occurred via other biting arthropods (mosquitoes and mites), but also through cannibalism, scavenging on carcasses, multiple use of syringes and needles, or ingestion of infective blood, droppings, or infected ticks [9]. Avian borreliosis is now considered an emerging disease globally [9,10].

Human diseases are caused by *Borrelia burgdorferi* sensu lato group (Bbsl) and less frequently by some members of the relapsing fever group borreliae. *Borrelia burgdorferi* strains acting as human pathogens have different conditions of propagation and reproduction, optimally reproducing in a laboratory at 33–37 °C and in semi-anaerobic environments, with *B. garinii* showing the highest temperature need [11]. This latter strain is present asymptomatically in birds, as reservoirs; moreover, birds have been shown to be capable of selectively amplifying it in experimental infections [12]. It has been observed that *B. garinii* can even generate an immune response in wild birds [13] and can cause mild symptoms in an experimental infection of birds [14].

The presence of human-pathogenic borreliae has been demonstrated in ticks collected from seabirds globally [15,16]. However, most investigations regarding *Borrelia burgdorferi* focus on testing the ticks attached to the birds [16,17,18,19,20,21,22,23]. A few sources prove the presence of Bbsl in the actual internal organs of wild birds, which was confirmed during necropsy [24]. Borrelia-naïve xenodiagnostic ticks could be infected by a bird with Bbsl group member *B. garinii* [25]. However, few experiments have been conducted with direct investigation of the living wild bird host for the most frequent human-pathogenic Bbsl group species [26,27,28,29].

Based on the above literature proving the infectivity of birds with Borreliae, we found it important to check direct blood samples of the captive wild birds for the presence of these blood-borne pathogens.

At the Wild Bird Hospital and Sanctuary Hortobágy, Hungary (later: WBHS), the main reason for requiring treatment is physical injury, which is 80% of the cases. The cause of the injury is often an electric shock by electrical wires. Both the infections acquired due to the injury and potential iatrogenic damage at the institution may contribute to complications, which may lead to the loss of the valuable animal, or the life-long need for care.

In this paper we are focusing on spirochetoid forms in the blood samples of the wild birds undergoing treatment at WBHS and on identifying the spirochetes as Borrelia species using specific immunostaining techniques.

## 2. Materials and Methods

### 2.1. Specimens

All samples were collected at the WBHS, at the request of the local veterinarian. For the direct inspection of its pathogen content, the CE-marked “DualDur kit” direct detection methodology was used. Two milliliters of venous blood was taken into 2 mL of an IVD CE-registered DualDur cell technology medium (registration according to EU regulations as an in vitro diagnostic device), pre-filled in Sarstedt syringes, mixed, and then stored at 4–8 °C. Samples were transported within 24 h at room temperature, prepared, and investigated within 172 h of phlebotomy. All samples were investigated with dark-field microscopy and 5 samples were also selected for immunofluorescence microscopy.

The wild bird subjects were 20 white storks (*Ciconia ciconia*, Linnaeus, 1758), 9 white-tailed eagles (*Haliaeetus albicilla*, Linnaeus, 1758), and 4 eastern imperial eagles (*Aquila heliaca*, Savigny, 1809), all hospitalized in the WBHS. We also investigated one of each of the common buzzard (*Buteo buteo*, Linnaeus, 1758), red-footed falcon (*Falco vespertinus*, Linnaeus, 1766), and rough-legged buzzard (*Buteo lagopus*, Swinhoe, 1861). The birds were not investigated for ticks or lice; however, no attached ticks were visibly present either during admission or during the blood draw.

DualDur cell technology medium contains, among others selective nutrients for *Borrelia burgdorferi*, glucose, human cell medium RPMI 1640, anticoagulant EGTA (ethylene glycol tetra acetic acid), and agents to stiffen the membrane of blood cells but not that of bacteria (e.g., caffeine salts, tetracaine), thus avoiding the decomposition of blood cells and the creation of artifacts such as pseudo-spirochetes.

### 2.2. Preparation Method: Borrelia Concentration

The Borrelia content of the samples was extracted using the DualDur kit and preparation method from [30]. The low-speed centrifugation (3300× *g*) separates the blood cells from the sample, then 2 mL of the supernatant plasma was subjected to high-speed centrifugation (15,500× *g*). After discarding the second supernatant, 2.5 µL of the resuspended sediment was examined under the dark-field microscopy setting described below.

### 2.3. Preparation Method: Immunofluorescent Staining

GeneTex *Borrelia burgdorferi* antibody FITC reagent (GTX36355, GeneTex, Inc., Irvine, CA, USA), which contains cleaned antibodies conjugated with fluorescein isothiocyanate (FITC) was used for the experiment. The specificity of the reagent was previously confirmed in a separate experiment using 3 different Borrelia strains as positive controls and one Treponema strain as a negative control (DSMZ, Braunschweig, Germany). The GeneTex reagent, original concentration 4–5 mg/mL, was further diluted to 1:40 in PBS and stored at 4–8 °C, and vortexed right before use for 2 min at 3500 rpm. Borrelia-containing sediment of the DualDur kit sample (prepared according to the Preparation method: Borrelia concentration) is mixed in a dark room at a ratio of 1:2 with the diluted GeneTex reagent solution, vortexed at 3500 rpm for 2 × 1 min, then incubated at 4–8 °C for 4 days. Right before the investigation, the sample is vortexed for one minute at 3500 rpm, and 2.5 µL of the sample is investigated.

### 2.4. Devices

A Nikon Ni-U microscope with a darkfield condenser and 60x objective, originally equipped with a halogen light source, was modified. Illumination with a 4 W Cree light emitting diode (LED, 450 nm wavelength) was applied with a corresponding driver and collimation device. LED power was adjusted between 1200 and 1500 mA [30].

For black and white pictures, a Basler MED acA3088-57um camera was used (Basler AG, Ahrensburg, Germany). Self-developed DualDur Diag recorder software was used with exposition times of 0.1–0.3 s with gain range of 5–18 (Basler Pylon viewer can be used instead, with the same settings).

Color pictures were taken with a Basler MED acA4112-30uc camera. A conventional green optical filter was placed in the phototube before the camera, and the excitation was performed via the dark-field illumination from a blue LED and eventual blue excitation light is filtered from the camera. Recording of the pictures of this camera was carried out with Basler Pylon viewer 6.3 software version (Basler AG, Ahrensburg, Germany), exposition time 0.4 s with gain range of 0.7–2.

### 2.5. Investigation

A total of 2.5 µL of the sample was put on a thin microscopy slide, covered with a thin cover slide of 18 × 18 mm (thickness #1, Epredia, Thermo Fisher Scientific, Waltham, MA, USA). Each case was investigated starting from the top left corner of the cover slide and going in an orderly manner towards the right, and then down and back left in a snake-like pattern. Steps of 3–5 mm were taken between two adjacent rows to avoid investigating the same objects again. In total, 6 rows were investigated, with 3 in each direction. High Borrelia burden was documented if >15 spirochetoid objects were found in the process, and low burden means <5.

**Figure 1:**
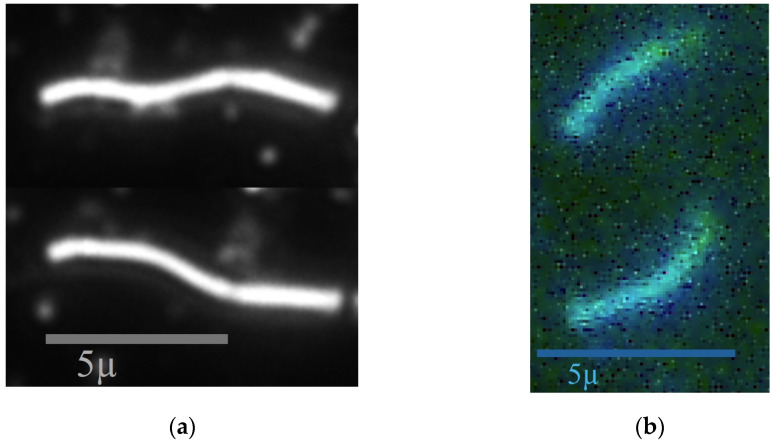
Representative images: (**a**) dark-field microscopy images of a medium-length spirochete in two typical phases of its motion; (**b**) fluorescence microscopy images of a live-stained short Borrelia in two typical phases of its motion.

Objects were photographed and selected. No other editing was carried out except for cutting the pictures for Figure 1. Since the total size of all videos and pictures is over 100 GB, a representative sample of the original pictures and videos were uploaded to Supplementary Material.

### 2.6. Statistical Analysis

Significance testing within the infectivity levels of the various groups was carried out with Fisher’s exact test methodology; significant differences were defined as *p* < 0.05. Each group was compared pairwise with each other group based on its infectivity level.

## 3. Results

To investigate blood-borne infections, 36 samples were taken from large-bodied wild birds who had been previously admitted to a bird hospital. The medical checkup at admission was carried out according to the normal routine of the hospital. The blood samples were collected and concentrated according to the DualDur methodology and were investigated via dark-field microscopy and immunofluorescence microscopy. The results concentrate on the findings relevant to spirochetal infections.

### 3.1. Clinical Status at Admission

The clinical investigation of the birds admitted to the hospital focused on finding the main reason for admittance and the exclusion of infectious diseases. Thus, the birds were not subjected to in-depth examination, especially because they had an evident injury. Later, if recovery was slower than expected, or new evident symptoms emerged, further examinations were requested or performed.

Half of the 36 samples came from patients that had an apparent injury, a broken or injured limb, in 10 out of 18 cases due to electric shock. All other areas of medicine were distributed evenly, and overall, only non-significant tendencies were observed: birds with physical injury had slightly less chance of a high or medium Borrelia count, whereas the typical areas of medicine where Borrelia infection may appear symptomatic are slightly overrepresented among the high to medium infection levels (neurology, rheumatology). Detailed numbers are outlined in Table 1.

Details of symptoms for all patients can be found in Supplementary Materials.

### 3.2. Laboratory Investigations

All samples were investigated with dark-field microscopy and five samples were also selected for immunofluorescence microscopy. In this article we will report the findings of the dark-field microscopy regarding only spirochetes and the specific immunofluorescence investigations with the anti-Borrelia-burgdorferi reagent. The results of the investigations are outlined in Table 2.

Fifteen out of twenty storks (75%) were infected with spirochetes, nine out of these (45%) had a high infection rate, five had a low, and one had a medium Borrelia burden.

Seven out of nine (78%) white-tailed eagles were infected with spirochetes, but only two (22%) had a high burden.

Three out of four (75%) eastern imperial eagles were infected.

Summing up the eagles, falcons, and buzzards, we conclude that out of the sixteen birds of prey whose habitat is not connected to surface waters, eleven (68%) had a spirochete infection, five (31%) with a high level, while storks had a 75% infectivity rate.

The differences within bird species or infectivity rate were non-significant.

The samples of two white-tailed eagles and three storks, having high Borrelia burden shown under the dark-field microscope, were investigated with immunofluorescence microscopy and confirmed to contain *Borrelia burgdorferi* sensu lato.

Representative images from the investigations are selected in Figure 1a (dark-field images) and Figure 1b (fluorescence images). Both show the motion of a spirochete. Figure 1a is the dark-field microscopy image of a medium-sized spirochete taken from two representative stages of its motion. Figure 1b is the fluorescence microscopy image of a live-stained short Borrelia that is still alive and showing a spirochetoid motion.

## 4. Discussion

### 4.1. Borrelia Quantity in the Blood and Other Tissues of the Host

Our objective was to investigate the presence of *B. anserina* in asymptomatic stages of avian borreliosis or reservoir-like presence of human pathogenic borreliae in blood.

Interestingly, both *B. anserina* and human pathogenic borreliae are controversially described as those that can only be detected in the blood in the early stages of infection [2]. According to publications, in most cases, *B. anserina* is permanently removed from the bird’s body, so after 7–17 days at the latest, the host animal will be asymptomatic and free of infection [2]. Whereas in human cases, it has already been repeatedly proven that *Borrelia burgdorferi* sensu lato can be detected using direct investigation methods throughout the entire course of infection from almost all tissues of the body, using PCR, microscope, culture, or xenodiagnosis [31,32,33,34,35].

Thus, the presence of Borrelia in the blood can be expected since the spread of infection is possible not only in symptomatic birds but also in birds that have already undergone infection or are asymptomatic carriers, also known as reservoirs [36,37,38]. Infection can even be reactivated by stress [4].

The problem of detectability of Borrelia in the blood of birds is likely dependent on the concentration of the bacteria in the blood, similar to the case of human infections. So, a difference by one or two orders of magnitude in bacteria density is possible in the case of the second-wave infection, or in reservoir-like presence [39,40]. The concentration method detailed in Methods was designed to identify scarce bacteria in the blood, increasing the concentration by two orders of magnitude.

### 4.2. The Specific Identification of Borrelia burgdorferi Sensu Lato vs. Borrelia anserina

The presence of the living, moving spirochetoid objects in the specimens was visually demonstrated by the use of the DualDur kit methodology. The specificity of the spirochetes as *Borrelia burgdorferi* sensu lato was determined by immunofluorescence microscopy. We will detail these statements below.

### 4.3. The Morphology of Various Genetic Traits of Borrelia in Various Media and in Various Circumstances

As with many bacteria, Borrelia also tend to adjust to the environment, but they are especially pleomorphic. The variety of shapes comes from the attachment and motion of flagella in the periplasmic space. The periplasmic space is the thin area between the cell wall complex (i.e., the cell cylinder) and the outer membrane. The spirochete shape is the result of a complex interaction between the cell cylinder and the periplasmic flagella. *B. burgdorferi* has a bundle of 7–11 helically shaped periplasmic flagella attached at each end of the cell cylinder and overlapping in the middle. This results in a flat-wave cell morphology [41]. However, this shape can vary according to the sampling procedure, the type of tissue, the preparation and staining methods, and by the type of investigation. Aberer and Duray performed microscopic investigations of numerous *Borrelia burgdorferi* cultures and observed structures varying from a long almost straight line with 2–4 waves to curly morphologies with up to 10 curls, some were even very short bacillus-like curved cells resembling vibrio bacteria or looked like two spirochetes joined “head to head”. Even leptospira-like morphologies were observed when the end of the Borrelia were bent [42]. Genetic changes can also cause morphological differences, sometimes very striking [41]. The exact shape and movement of Borrelia extracted from human or animal samples with the DualDur kit resemble the recent pictures and videos of Borrelia taken with FISH technique and confocal microscopy in capillaries [43,44].

The morphological description of *B. anserina* in the literature is different from the shape of the human pathogenic Bbsl; however, we must take into consideration that the shape in a direct sample will also be different in the DualDur kit, just as the pictures of a naturally occurring Bbsl group spirochete are different from the ones derived from a cell culture, seen after several passages in the culture medium. We can still conclude that *B. anserina* is longer than Bbsl group spirochetes, 9–21 µm compared to 7–17 µm, so the two could be differentiated. In the investigations, we mostly saw short, medium, and long spirochetes, but only sometimes did we see very long moving spirochetes. There was no attempt to determine the exact species based on length.

### 4.4. Specificity of the Immunofluorescent Reagent

GeneTex GTX36355 immunofluorescence reagent was tested at Synlab laboratories, Hungary, for use in this specific setting. This reagent contains a whole cell preparation from *B. burgdorferi* purified by protein A chromatography or sequential differential precipitations. The official documentation at the time of purchase did not contain any information on the cross-reactions, so specificity was tested on 3 *Borrelia burgdorferi* sensu lato strains and one *Treponema* spirochete available from DSMZ Germany. Only the Bbsl strains were reactive.

Since a cultivable *B. anserina* strain was not readily available from a biobank, we had to check the potential cross-reactions between Bbsl and *B. anserina* that could hinder the differentiation of the two types of Borrelia using the immunofluorescent reagent. The antigenic properties of the two groups are markedly different, as shown by the SDS-PAGE separation of the antigenic proteins. Despite that, antigenic cross-reactivity was confirmed between the p41 flagellin (41 kD) region of Bbsl and the 39 kD flagellin region of *B. anserina*. It was however observed that the anti-flagellin antibodies did not bind to all regions of the flagellins [45], and therefore, the cross-reaction would cause a partial fluorescence activity if the anti-flagellin antibodies in the Bbsl reagent were bound to the *B. anserina* flagellins. Since the pictures and videos presented here show full coverage of the spirochetoid forms, the reagent was specifically binding to the proteins of *B. burgdorferi*.

### 4.5. Our Results in the View of Previous Literature

Both the causative agents of avian and human borreliosis, *Borrelia anserina,* and *Borrelia burgdorferi* sensu lato have been mostly investigated in the ticks feeding on wild birds and not in the actual blood samples of wild birds. Only a few publications have demonstrated the presence of borreliae in blood samples of living wild birds.

The genome of a relapsing-fever type Borrelia, similar to *Borrelia anserina* (<93% sequence homology), was found in one red-tailed hawk out of the twenty-three wild birds investigated [6]. One in thirty-nine blood samples of small-bodied passerine birds showed the presence of *Borrelia burgdorferi* (Bbsl) in cultivation and then confirmed in PCR [28]. Of 1254 small-bodied passerine birds, 4.2% yielded a positive blood sample with Bbsl-nested PCR [29]. Two cited publications provide an example of using xenodiagnostic ticks to prove the presence of *Borrelia burgdorferi* sensu lato in the body of wild birds. Three out of three [26] and four out of five [27] small-bodied wild birds could infect tick larvae feeding on them in the laboratory experiment. PCR and cultivation are known to have very low sensitivity in investigating real-life blood samples [46], and in this sense, the small-base xenodiagnostic investigations could be more in line with reality. In any case, the very low prevalence of Borrelia according to PCR could not easily explain the high level of xenodiagnostic success and the rate of Borrelia presence in the samples of our research with the novel concentration and examination method. Albeit on a small base and not a prospective sample of wild birds, our results provide hope that novel direct investigations will be able to further prove or disprove the theory that *Borrelia burgdorferi* may have a life cycle that involves wild birds as reservoirs.

### 4.6. Future Outlook and Limitations of the Study

This study of captive birds of prey and storks shows a high infectivity rate; however, it has no base of healthy birds to compare this group to. The reason is that the primary objective of the investigations was to help with the diagnosis of the birds that were admitted to the Wild Bird Hospital and Sanctuary; thus, there was no “negative group” of healthy birds to compare to.

More research is needed to confirm the different species of Borrelia found in blood samples of wild birds, for example, by nucleic acid amplification tests or full genome sequencing. Further examinations during the admission to the WBHS are necessary to define whether any other symptoms can be detected besides the apparent injury to find out if any pre-existing infection may have led to the wild birds acquiring that injury. A prospective study with an appropriate base, involving other countries, is necessary to determine the difference in Borrelia infection between the general population of wild birds by species and those admitted to the hospital for various reasons.

## 5. Conclusions

The causative agent of avian borreliosis, *Borrelia anserina*, and that of human Lyme borreliosis, *Borrelia burgdorferi* sensu lato can be present in various wild birds across the globe as a reservoir, even if the carrying birds only become symptomatic in special circumstances. Ticks can become infected by feeding on the blood of the reservoir host, and this precludes the presence of Borrelia in the blood which the ticks feed on. We have found based on direct blood samples investigated with the DualDur kit at the Wild Bird Hospital and Sanctuary that out of the sixteen birds of prey, eleven (68%), and fifteen out of twenty storks (75%) were infected with spirochetes. Some of these spirochetes were confirmed morphologically and via immunofluorescence microscopy to be the human pathogenic *Borrelia burgdorferi* sensu lato. The birds to be investigated were selected by the veterinarian of the Hospital, and therefore had a health issue; thus, they cannot be considered a representative sample of the free wild birds. The infectivity rate of the same species of wild birds may be different if selected randomly from the free population. The low base and the selection bias are limiting factors of this study. No correlation was found between the primary symptoms as a reason for admission to the hospital and the level of Borrelia burden found in the laboratory.

## Figures and Tables

**Table 1:** Distribution of the level of infectivity among areas of medicine for the leading symptom at admission. All differences between infectivity groups are pairwise non-significant.

	Borrelia Burden		
Area of Medicine for Leading Symptom	High to Medium	Low to None	Total
Epidemiology	1	1	2
Internal medicine	1	1	2
Nestling pathology	4	3	7
Neurology	2	1	3
Toxicology	0	1	1
Rheumatology	2	1	3
Emergency veterinary medicine	8	10	18
Grand Total	18	18	36

**Table 2:** Distribution of the level of infectivity among wild bird species. All differences between infectivity groups are pairwise non-significant.

Species	Borrelia Burden by Level	Percentage of Species
Eastern imperial eagle (*Aquila heliaca*)	4	11%
high	2	50%
medium	1	25%
none	1	25%
Common buzzard (*Buteo buteo*)	1	3%
none	1	100%
Rough-legged buzzard (*Buteo lagopus*)	1	3%
none	1	100%
White stork (*Ciconia ciconia*)	20	56%
high	9	45%
medium	1	5%
low	5	25%
none	5	25%
Red-footed falcon (*Falco vespertinus*)	1	3%
high	1	100%
White-tailed eagle (*Haliaeetus albicilla*)	9	25%
high	2	22%
medium	2	22%
low	3	33%
none	2	22%
Total	36	100%

## Data Availability

The original contributions presented in the study are included in the article/Supplementary Material; further inquiries can be directed to the corresponding author.

## Supplementary Materials

The following supporting information can be downloaded at: https://www.mdpi.com/article/10.3390/ani14243553/s1.
