# Supplementary figures and images for: Presence of Borrelia Spirochetes in White Stork (Ciconia ciconia), White-Tailed Eagle (Haliaeetus albicilla), and Eastern Imperial Eagle (Aquila heliaca): Hospitalized in a Wild Bird Hospital and Sanctuary (Hortobágy, Hungary)
Source: Animals (Basel). 2024 Dec 10;14(24):3553. doi: 10.3390/ani14243553 (PMC11672504; doi:10.3390/ani14243553)

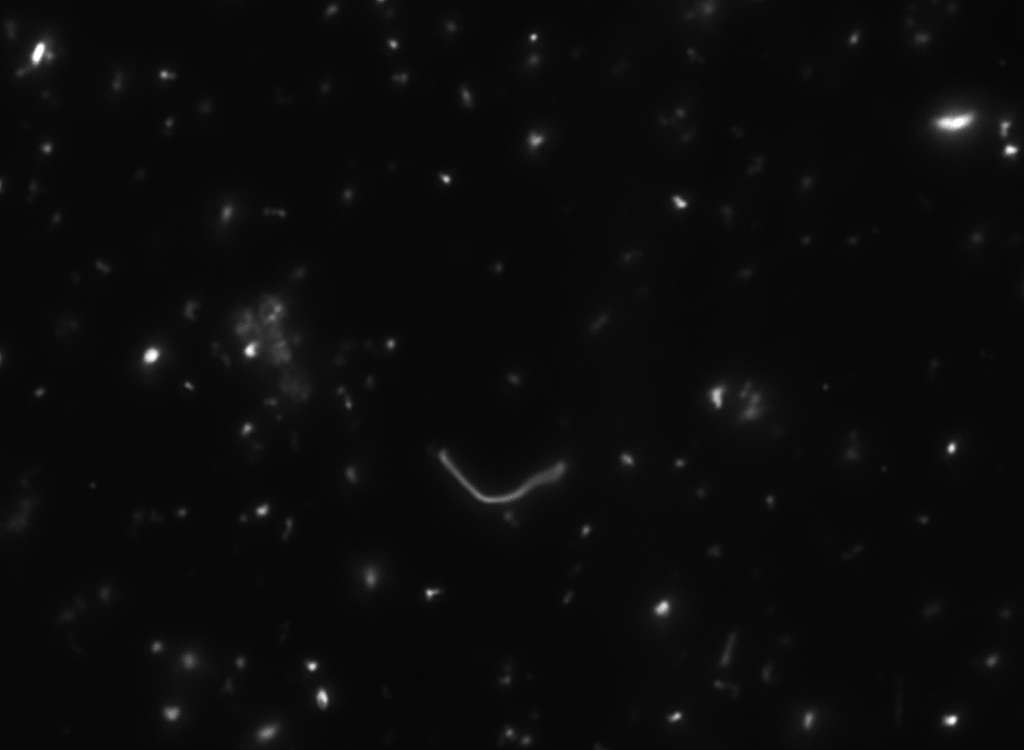

Supplement: Supplementary file 1 [file animals-14-03553-s001.zip › All additional data and pictures/Pistures and videos/15917_2023-08-22-19-45-19.png]

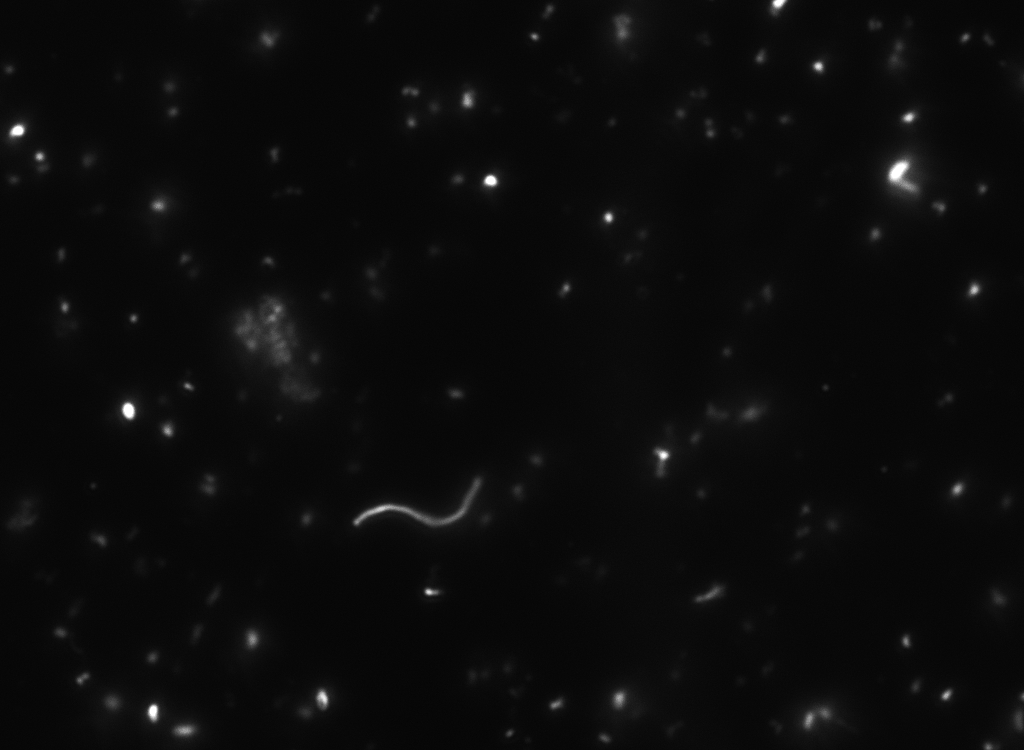

Supplement: Supplementary file 1 [file animals-14-03553-s001.zip › All additional data and pictures/Pistures and videos/15917_2023-08-22-19-45-23.png]

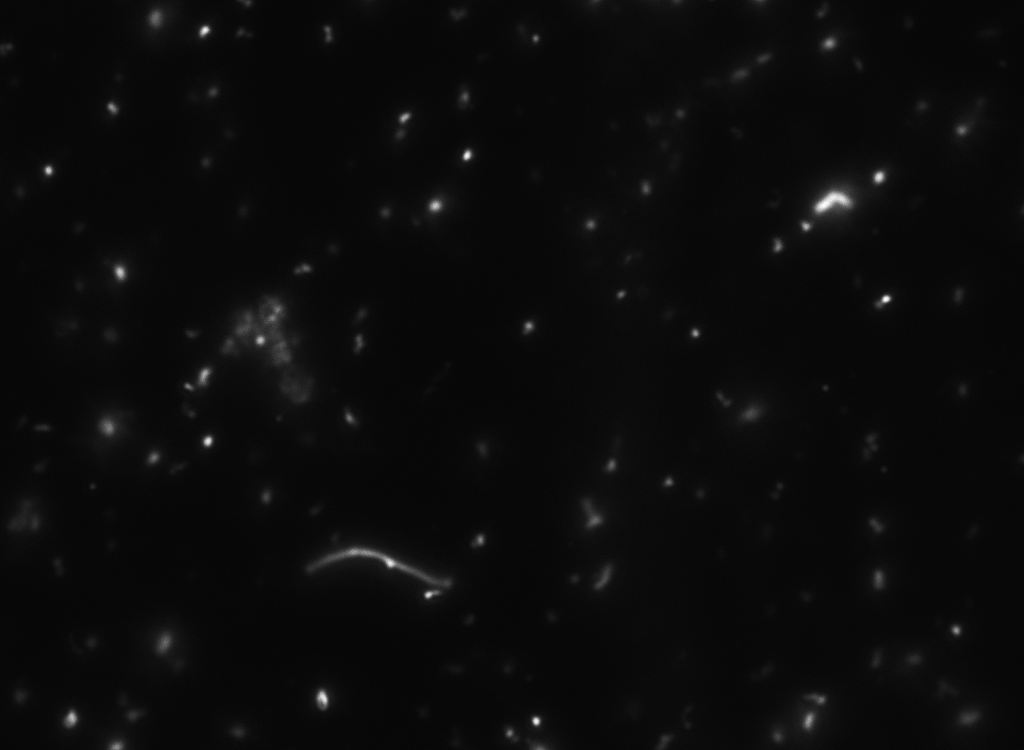

Supplement: Supplementary file 1 [file animals-14-03553-s001.zip › All additional data and pictures/Pistures and videos/15917_2023-08-22-19-45-26.png]

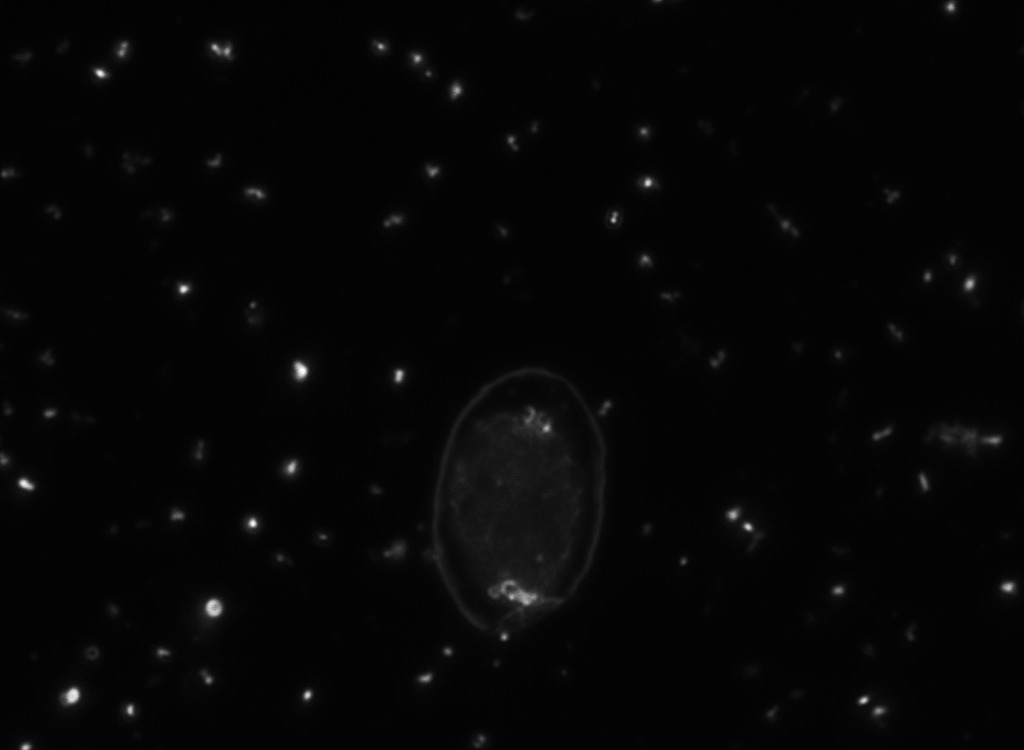

Supplement: Supplementary file 1 [file animals-14-03553-s001.zip › All additional data and pictures/Pistures and videos/15917_2023-08-22-19-48-55.png]

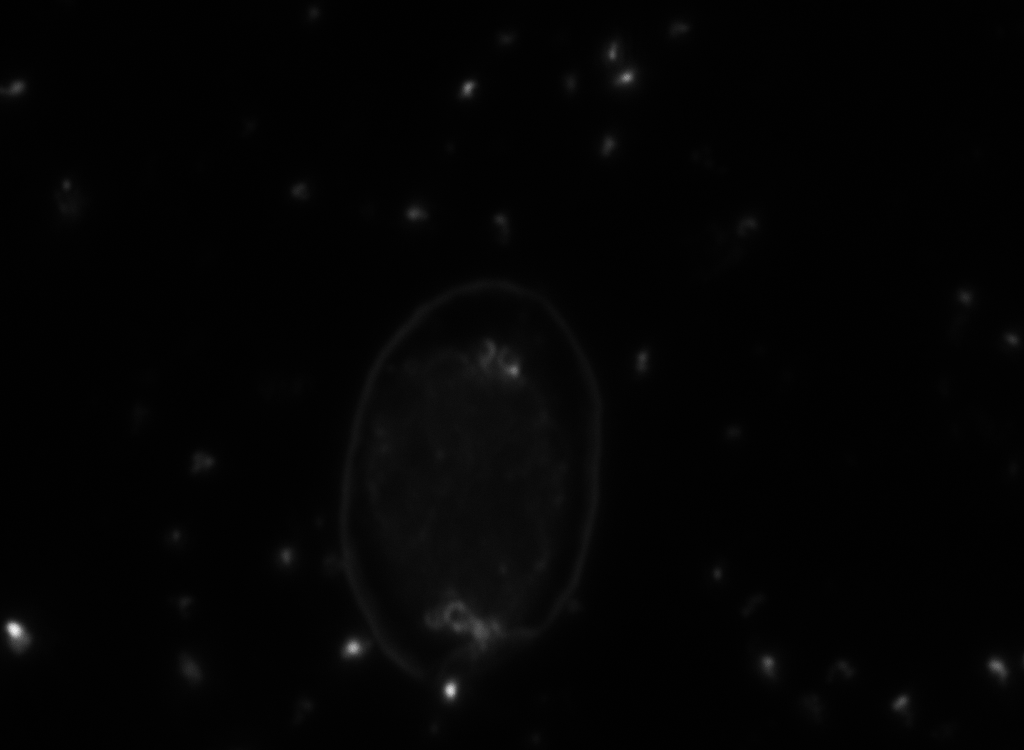

Supplement: Supplementary file 1 [file animals-14-03553-s001.zip › All additional data and pictures/Pistures and videos/15917_2023-08-22-19-49-18.png]

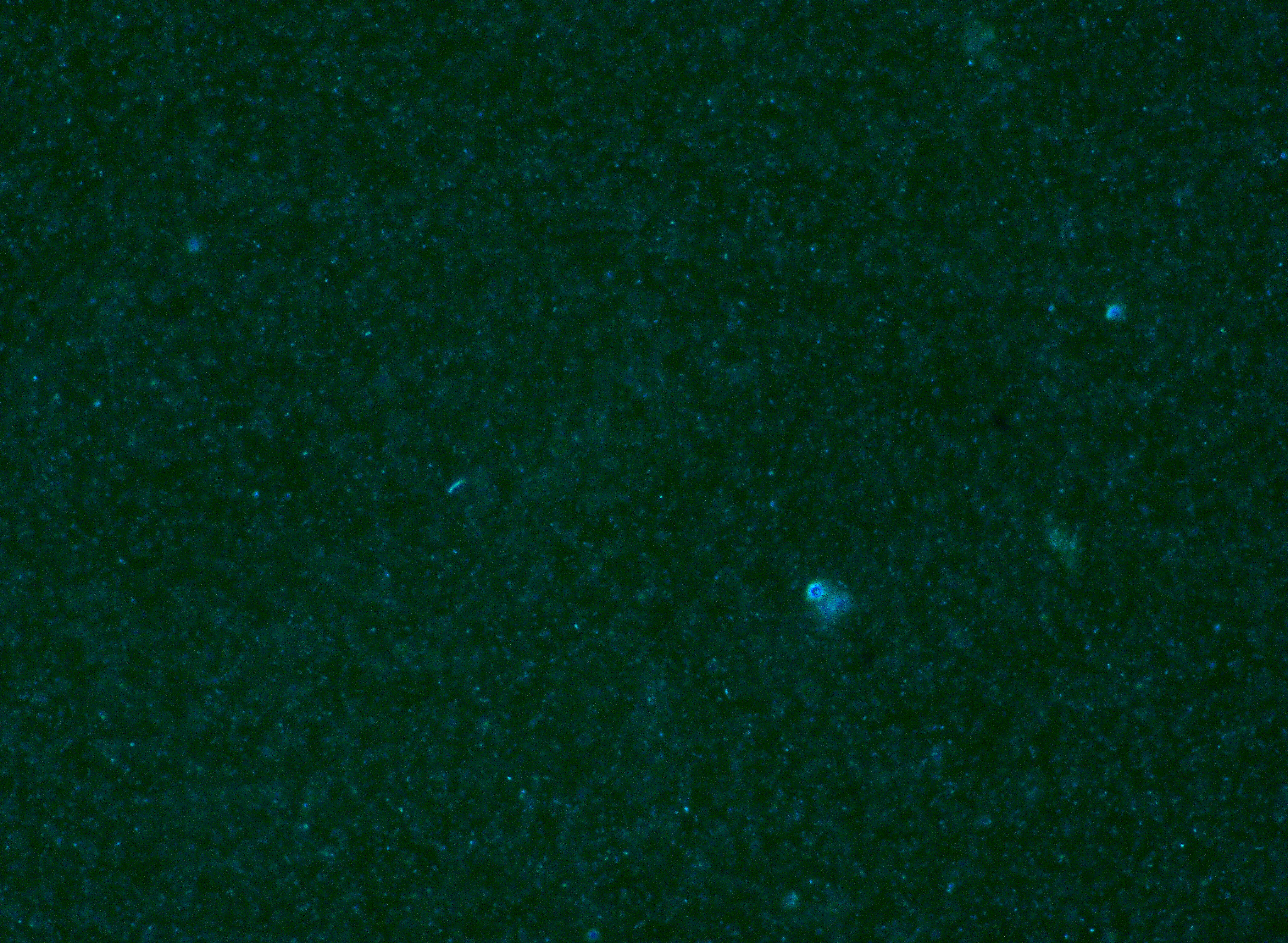

Supplement: Supplementary file 1 [file animals-14-03553-s001.zip › All additional data and pictures/Pistures and videos/Basler_acA4112-30ucMED__40031682__20230710_174524442_0133.tiff]

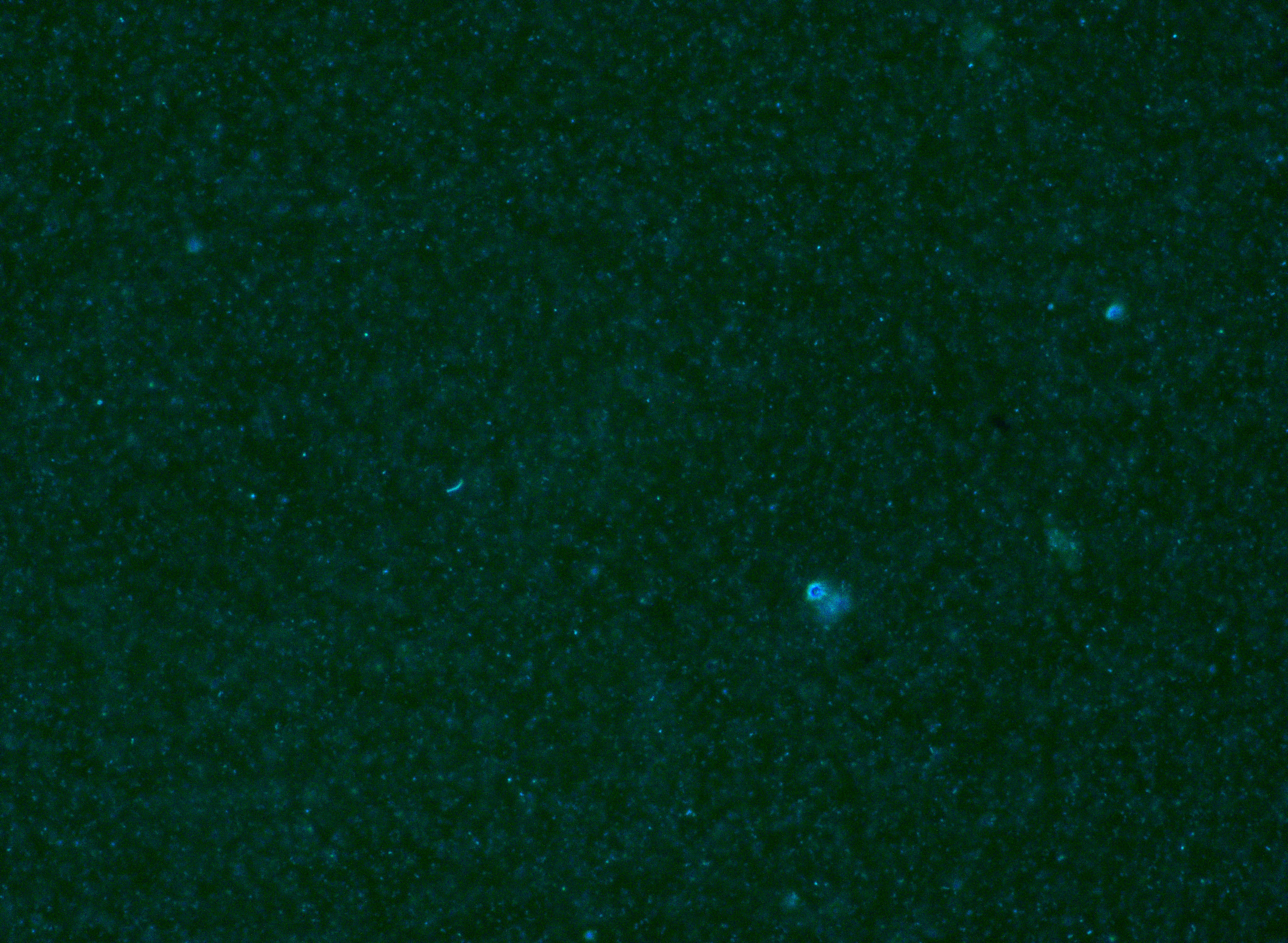

Supplement: Supplementary file 1 [file animals-14-03553-s001.zip › All additional data and pictures/Pistures and videos/Basler_acA4112-30ucMED__40031682__20230710_174524442_0134.tiff]

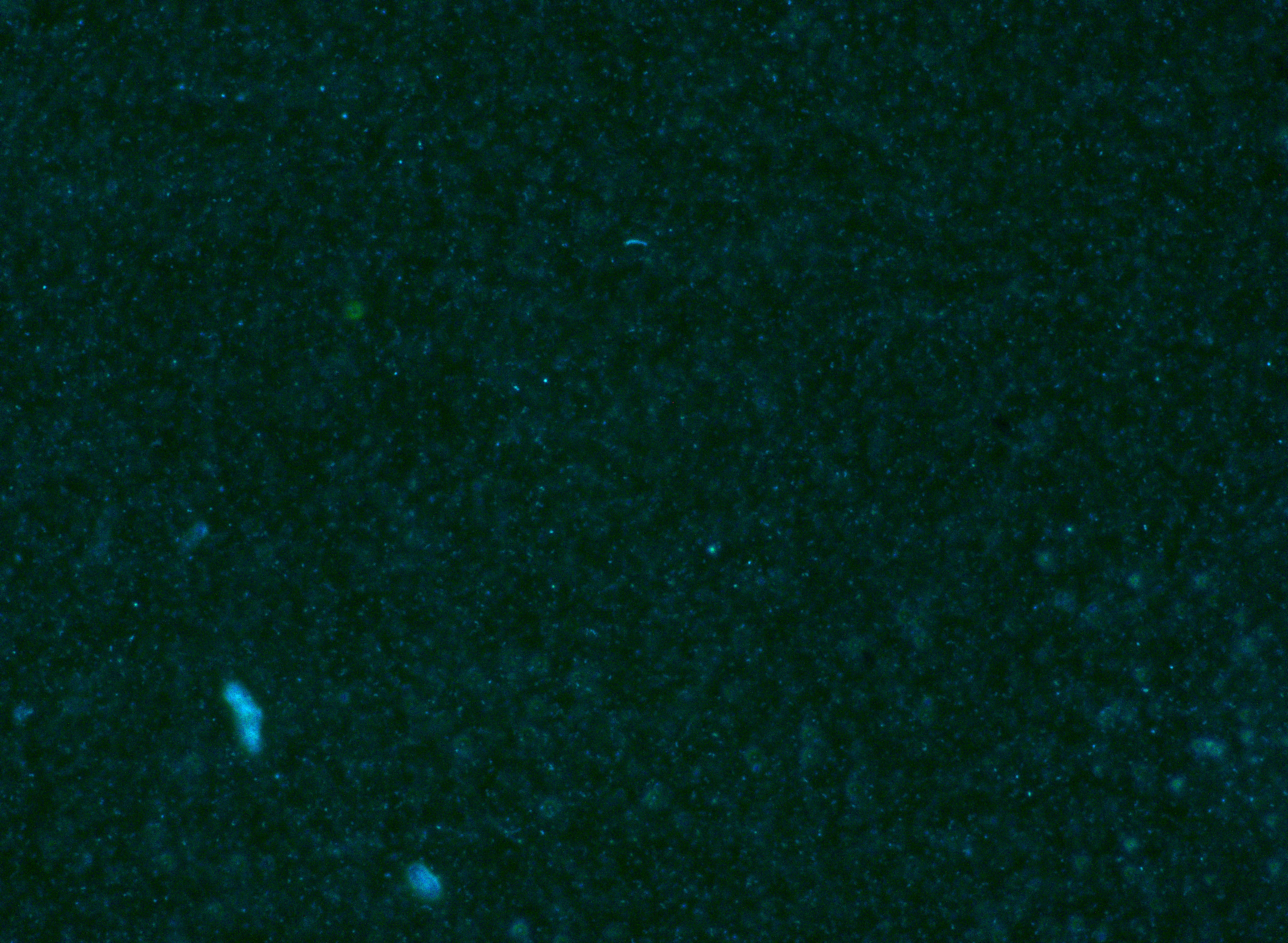

Supplement: Supplementary file 1 [file animals-14-03553-s001.zip › All additional data and pictures/Pistures and videos/Basler_acA4112-30ucMED__40031682__20230710_174634400_0130.tiff]

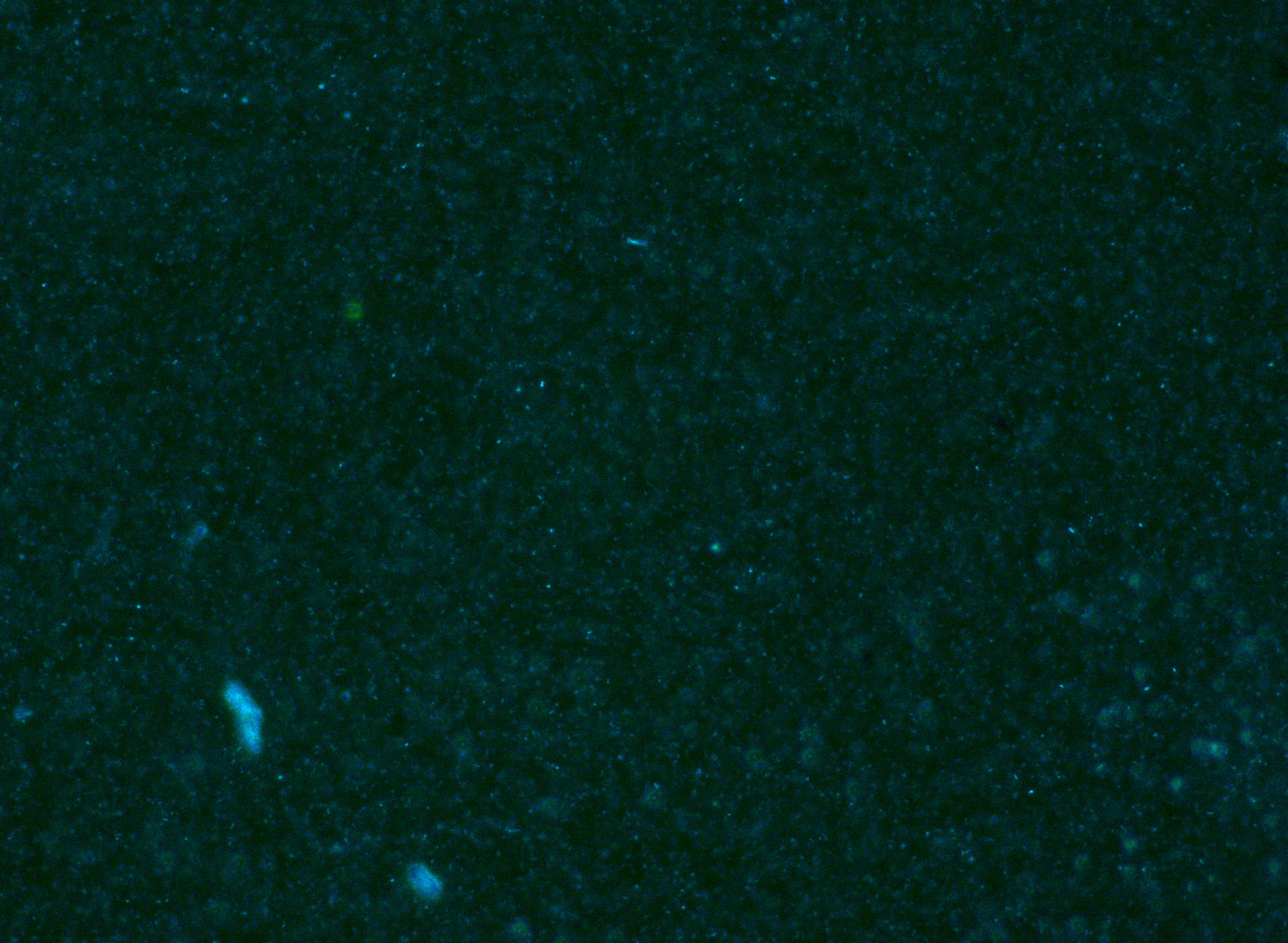

Supplement: Supplementary file 1 [file animals-14-03553-s001.zip › All additional data and pictures/Pistures and videos/Basler_acA4112-30ucMED__40031682__20230710_174634400_0131.tiff]
